# Ganglioglioma with adverse clinical outcome and atypical histopathological features were defined by alterations in *PTPN11/KRAS/NF1* and other RAS-/MAP-Kinase pathway genes

**DOI:** 10.1007/s00401-023-02561-5

**Published:** 2023-03-27

**Authors:** Lucas Hoffmann, Roland Coras, Katja Kobow, Javier A. López-Rivera, Dennis Lal, Costin Leu, Imad Najm, Peter Nürnberg, Jochen Herms, Patrick N. Harter, Christian G. Bien, Thilo Kalbhenn, Markus Müller, Tom Pieper, Till Hartlieb, Manfred Kudernatsch, Hajo Hamer, Sebastian Brandner, Karl Rössler, Ingmar Blümcke, Samir Jabari

**Affiliations:** 1 Department of Neuropathology, Partner of the European Reference Network (ERN) EpiCARE, Universitätsklinikum Erlangen, FAU Erlangen-Nürnberg, Erlangen, 91054 Germany; 2 Department of Molecular Medicine, Cleveland Clinic Lerner College of Medicine, Case Western Reserve University, Cleveland, USA; 3 Genomic Medicine Institute, Lerner Research Institute, Cleveland Clinic, Cleveland, OH 44195 USA; 4 Charles Shor Epilepsy Center, Neurological Institute, Cleveland Clinic, Cleveland, USA; 5 Stanley Center for Psychiatric Research, Broad Institute of Harvard and M.I.T, Cambridge, MA 02142 USA; 6 Cologne Center for Genomics (CCG), Medical Faculty of the University of Cologne, University Hospital of Cologne, 50931 Cologne, Germany; 7 Center for Neuropathology and Prion Research, LMU Munich, Munich, Germany; 8 Department of Epileptology (Krankenhaus Mara), Medical School, Bielefeld University, Bielefeld, 33617 Germany; 9 Center for Pediatric Neurology, Neurorehabilitation, and Epileptology, Schoen-Clinic, Vogtareuth, 83569 Rosenheim, Germany; 10 Epilepsy Center, EpiCARE Partner, Universitätsklinikum Erlangen, FAU Erlangen-Nürnberg, Erlangen, 91054 Germany; 11 Department of Neurosurgery, EpiCARE Partner, Universitätsklinikum Erlangen, FAU Erlangen-Nürnberg, Erlangen, Germany; 12 Department of Neurosurgery, EpiCARE Partner, Medical University of Vienna, Vienna General Hospital, Vienna, Austria; 13 Department of Neurosurgery (Evangelisches Klinikum Bethel), Medical School, Bielefeld University, Bielefeld, 33617 Germany

**Keywords:** Brain, Seizure, Summary plots, Epilepsy, DNA methylation

## Abstract

Exome-wide sequencing studies recently described *PTPN11* as a novel brain somatic epilepsy gene. In contrast, germline mutations of *PTPN11* are known to cause Noonan syndrome, a multisystem disorder characterized by abnormal facial features, developmental delay, and sporadically, also brain tumors. Herein, we performed a deep phenotype-genotype analysis of a comprehensive series of ganglioglioma (GG) with brain somatic alterations of the *PTPN11/KRAS/NF1* genes compared to GG with common MAP-Kinase signaling pathway alterations, i.e., *BRAFV600E*. Seventy-two GG were submitted to whole exome sequencing and genotyping and 84 low grade epilepsy associated tumors (LEAT) to DNA-methylation analysis. In 28 tumours, both analyses were available from the same sample. Clinical data were retrieved from hospital files including disease onset, age at surgery, brain localization, and seizure outcome. A comprehensive histopathology staining panel was available in all cases. We identified eight GG with *PTPN11* alterations, copy number variant (CNV) gains of chromosome 12, and the commonality of additional CNV gains in *NF1, KRAS*, *FGFR4* and *RHEB*, as well as *BRAFV600E* alterations. Histopathology revealed an atypical glio-neuronal phenotype with subarachnoidal tumor spread and large, pleomorphic, and multinuclear cellular features. Only three out of eight patients with GG and *PTPN11/KRAS/NF1* alterations were free of disabling-seizures 2 years after surgery (38% had Engel I). This was remarkably different from our series of GG with only *BRAFV600E* mutations (85% had Engel I). Unsupervised cluster analysis of DNA methylation arrays separated these tumours from well-established LEAT categories. Our data point to a subgroup of GG with cellular atypia in glial and neuronal cell components, adverse postsurgical outcome, and genetically characterized by complex alterations in *PTPN11* and other *RAS-/MAP*-Kinase and/or mTOR signaling pathways. These findings need prospective validation in clinical practice as they argue for an adaptation of the WHO grading system in developmental, glio-neuronal tumors associated with early onset focal epilepsy.

**Supplementary Information:**

The online version contains supplementary material available at 10.1007/s00401-023-02561-5.

## Introduction

Protein Tyrosine Phosphatase Non-receptor Type 11 (*PTPN11*) has been recently discovered as a new candidate gene in brain tissue obtained from drug-resistant structural epilepsies [2, 18, 23]. The *PTPN11* gene encodes for an early non-receptor-type protein tyrosine phosphatase SHP-2 (src homology region 2-domain phosphatase-2) of the RAS-/MAP-Kinase pathway. Germline variants in *PTPN11*, e.g., missense variants or copy number variations (CNV), or other *RAS-/MAP*-Kinase signaling pathway genes including *SHOC2*, *CBL*, *KRAS*, are known to cause an autosomal dominant multisystem disorder—the Noonan syndrome—characterized by several non-central nervous system (CNS) disorders, including cardiovascular abnormalities, lymphatic abnormalities, and growth hormone deficiencies [10, 27, 30, 32]. Thirteen percent of patients with Noonan syndrome also have recurrent seizures and sporadically develop glial and glio-neuronal brain tumors, e.g., pilocytic astrocytoma and dysembryoplastic neuroepithelial tumors (DNT) [27, 30, 31].

We recently identified an accumulation of brain somatic *PTPN11* alterations in low-grade epilepsy-associated brain tumors (LEAT) [23], the second largest lesion category in drug-resistant focal epilepsies amenable to neurosurgical treatment [6, 21]. Ganglioglioma (GG) account for approx. 64% of all LEAT, 82% of which affect the temporal lobe, and histopathologically classified as WHO CNS grade 1 [33]. 80% of these patients become free from disabling seizures, i.e., 5 years after surgery, with many patients also stopping their anti-seizure medication [21]. Nevertheless, no biomarker is available for the remaining 20% of patients who do not benefit from a tailored epilepsy surgery approach.

We addressed this issue by studying a comprehensive cohort of GG with whole-exome sequencing, DNA methylation, histopathology, and clinical outcome parameter. Interestingly, our analysis identified a group of GG with complex alterations in *PTPN11*/*KRAS*/*NF1* and other RAS-/MAP-Kinase or mTOR pathway genes, adverse clinical outcome, and atypical histopathological features.

## Methods

One-hundred-twenty-eight samples of histopathologically confirmed LEAT and a pre-defined set of clinical features were collected at the University Hospital in Erlangen, Germany; Klinikum Bethel-Mara, Bielefeld University, Germany; and Schoen-Klinik Vogtareuth, Germany. Seventy-two samples were snap frozen at − 80 °C and submitted to whole-exome sequencing and Single-Nucleotide-Polymorphism analysis (SNP) using the Global Screening Array with Multi-disease drop-in (v1.0; Illumina, San Diego, CA, USA) as described elsewhere [23]. Eighty-four samples were formalin-fixed and paraffin-embedded (FFPE) and submitted to DNA methylation analysis using 450K and 850K arrays (Illumina, California, USA). Whole exome sequencing, SNP array, and DNA methylation analysis were jointly available in 28 samples (Table 1). The Ethics Committee of the Medical Faculty of the Friedrich-Alexander-University (FAU) Erlangen-Nürnberg, Germany, approved this study within the framework of the EU project “DESIRE” (FP7, grant agreement #602,531; AZ 92_14B) and European Reference Network EpiCARE” (grant agreement #769,051; AZ 193_18B).

**Table 1:**
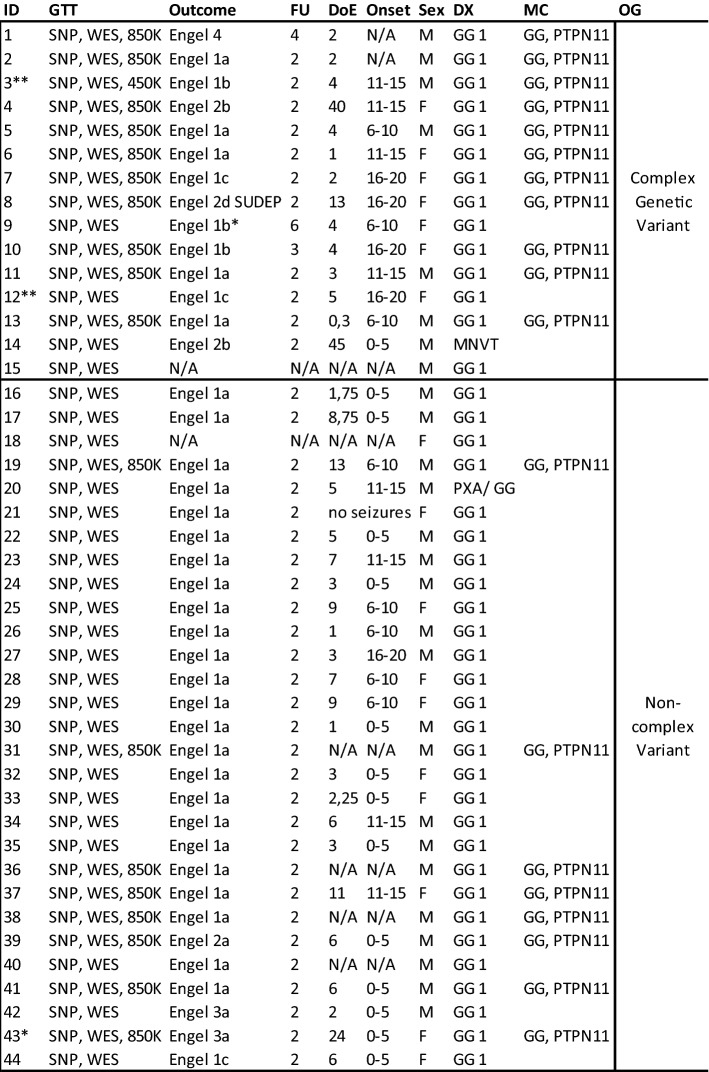

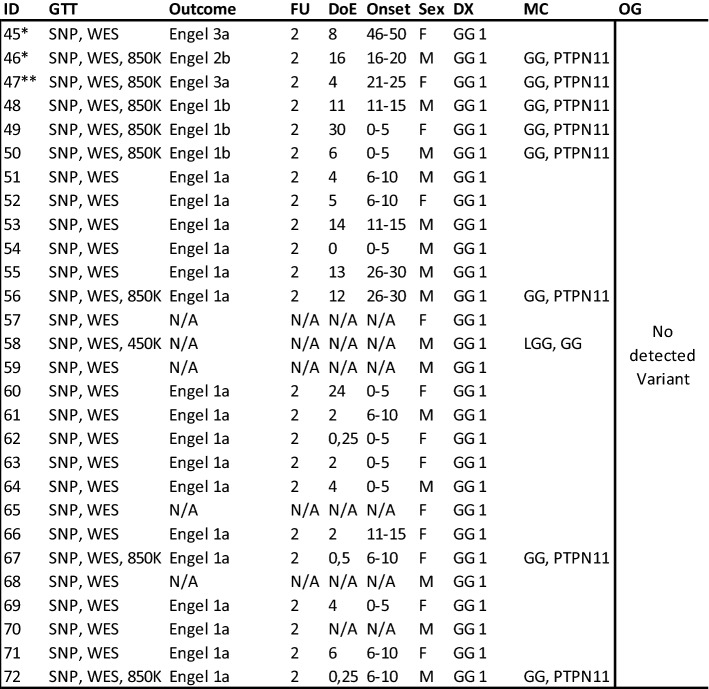
Summary of 72 patients with WES and SNP, 28 of which also had DNA methylation analysis

**Fig. 1:**
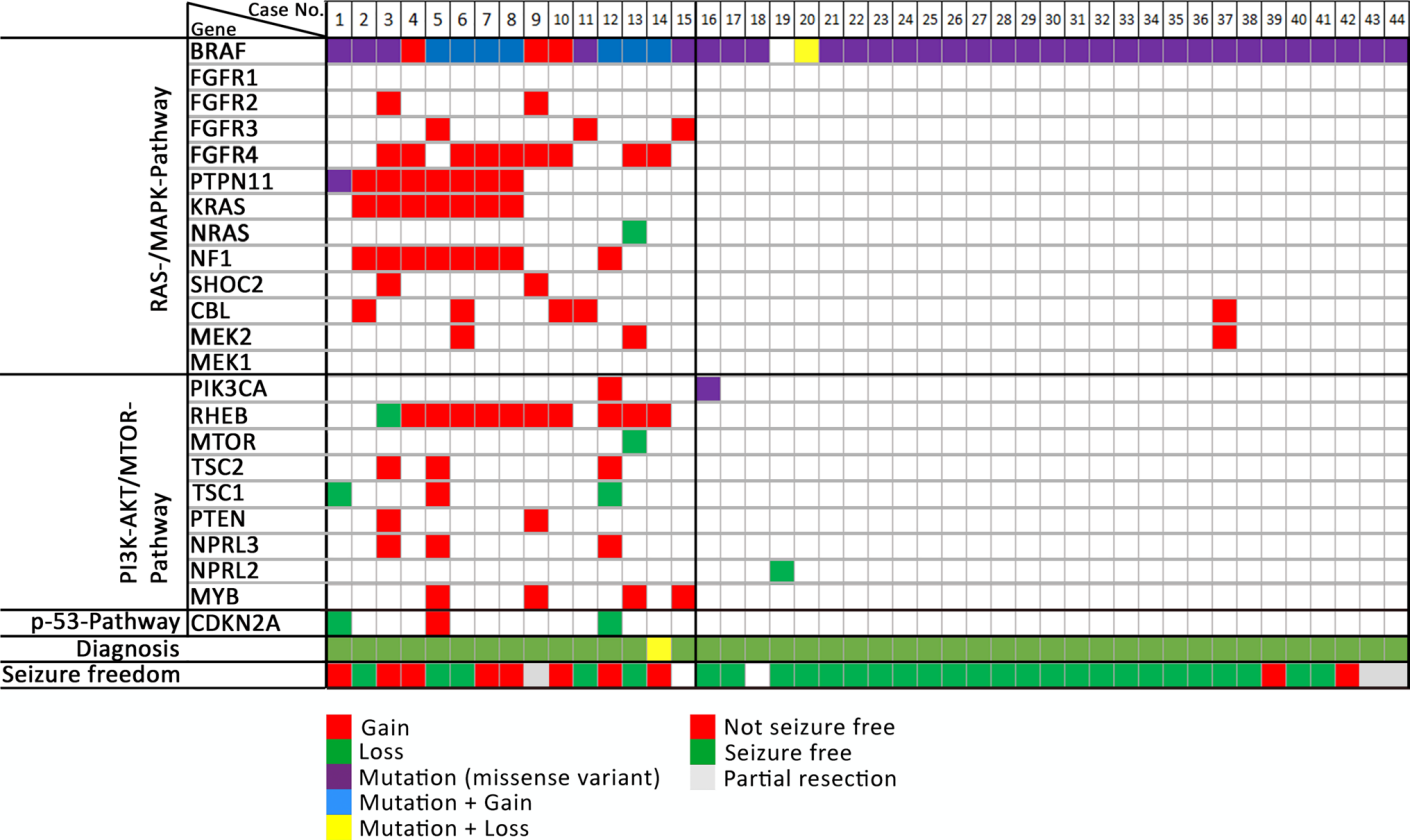
Oncoplot of WES and CNV detection. Each column represents a patient sample submitted to WES (same ID as in Table 1). We have intentionally classified these data into three groups of patients: GG with complex genetic variants (cases 1–15), GG with non-complex genetic variants (cases 16–44), and GG without detected variants (cases 45–72, not shown). Diagnosis: Histopathology review confirmed the diagnosis of ganglioglioma (green tiles) in 43 cases. Case #14 was compatible with the diagnosis of MNVT (yellow tile). Seizure freedom was defined by Engel’s outcome class 1 [21]. Lack of seizure freedom was explicable by incomplete resection in cases #9, #42, and #43 (greyish tiles). Outcome data was missing in cases #15 and #18 (white tiles)

**Fig. 2:**
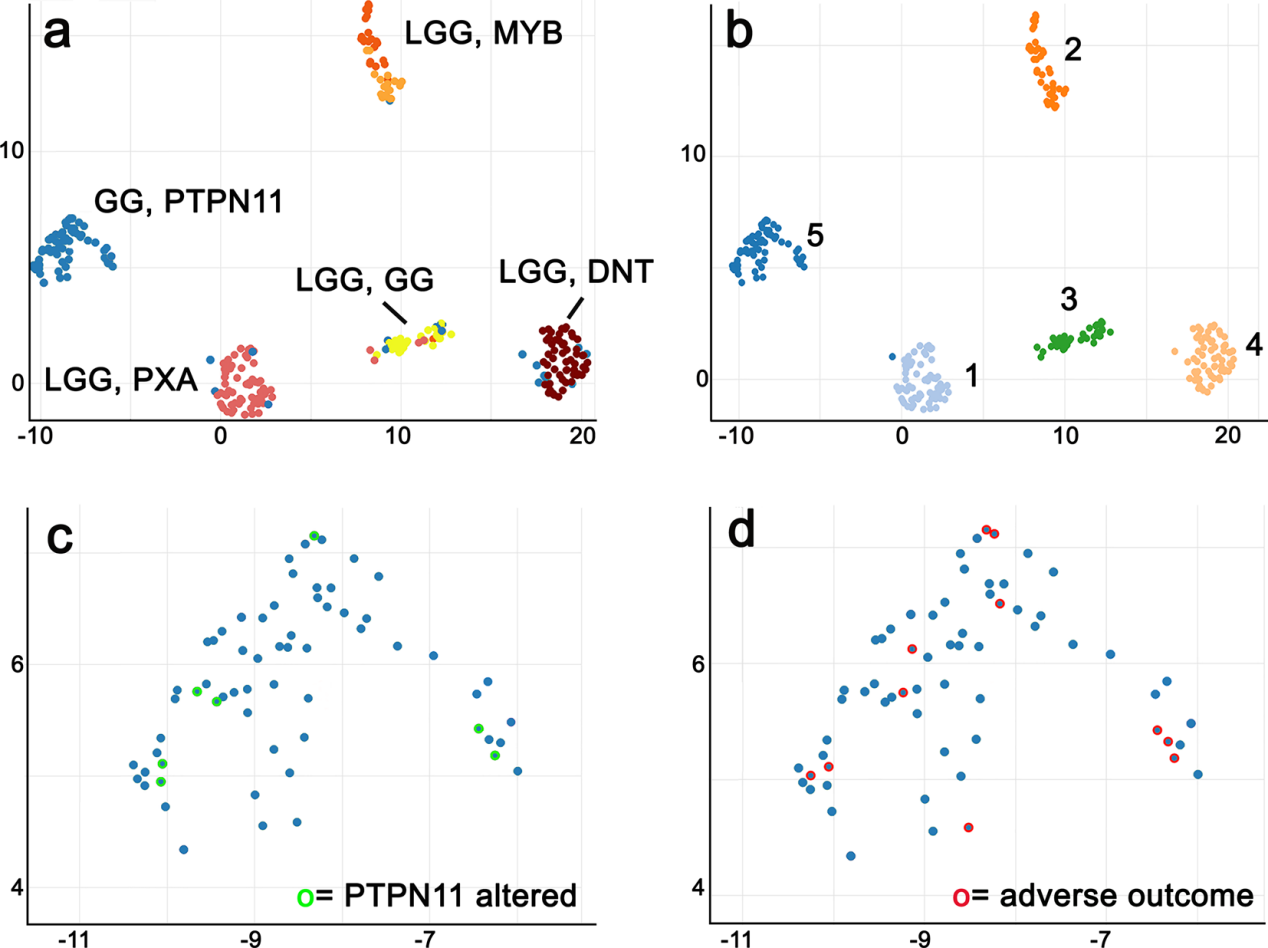
Unsupervised DNA methylation clustering defined five LEAT subgroups. UMAP-clustering (**a**) of methylation data revealed a novel methylation class distinct from previously recognized groups (*GG, PTPN11* = GG with adverse outcome and *PTPN11/KRAS/NF1 alterations*; *n* = 63). The reference groups were annotated as low-grade glioma, Ganglioglioma (LGG, GG, *n* = 26 in yellow), Dysembryoplastic Neuroepithelial Tumour (LGG, DNT, *n* = 56 in brown), Pleomorphic Xantoastrocytoma (LGG, PXA, *n* = 63 in pinkish) and diffuse astrocytoma MYB- or MYB-L1 altered (LGG, MYB, *n* = 45 in orange) by Capper et al. 2018 and Wefers et al. 2020. All new cases described in this study were labeled in blue (see Table 1, supplemental table, online resource). Unsupervised clustering using HDBSCAN (**b**) confirmed the novel DNA methylation class (class 5 on the left). All eight samples with *PTPN11/KRAS/NF1* alterations were assigned to this group by HDBSCAN (green open circles in **c**, dark blue dot in close proximity to group 1 in **b**). Eleven out of 19 patients not being seizure-free were also assigned to this group (red open circles in **d;** patients with partial resection were not visualized)

ID = case numbers, GTT = Genetic testing tool using either Single Nucleotide Polymorphism (SNP), Whole-Exome Sequencing (WES), 850K or 450K DNA methylation arrays; Outcome = most recent postsurgical outcome, i.e., seizure freedom, according to Engel [21]; N/A = not available; Follow-up in years; DoE. = Duration of Epilepsy; Onset = disease onset (age in years); Sex: F—female, M = male; DX = histopathology diagnosis: Ganglioglioma CNS WHO grade 1 (GG 1), Ganglioglioma analogue CNS WHO grade 2 (GG 2), Ganglioglioma analogue CNS WHO grade 3 (GG 3), composite pleomorphic xanthoastrocytoma with ganglioglioma (PXA/GG), multinucleated vacuolated tumor (MNVT). MC – methylation classes: Ganglioglioma with adverse clinical outcome (GG, PTPN11), Ganglioglioma (LGG, GG), Pleomorphic Xantoastrocytoma (LGG, PXA), (also see Fig. 2); Oncoplot Group (OG) = genetic subgroups from WES/SNP-array (see also Fig. 1). Sixty-two cases were located in the temporal lobe, cases #10, #17, #23, #43 and #50 in the parietal lobe, cases #41, #46 and #67 in the frontal lobe, case #24 in the occipital lobe and case #15 had no localizing data

*Adverse outcome due to incomplete resection

**Patient received a second surgery due to lack of seizure freedom

### Histology and immunohistochemistry

FFPE tissue blocks and glass slides were retrieved from the archives of the Neuropathological Institute at University Hospital Erlangen. Hematoxylin and Eosin stainings were available from all blocks and microscopically examined by two senior authors and experienced neuropathologists (IB, RC). Additional immunohistochemical stainings were performed with the Ventana BenchMark ULTRA Immunostainer and the OptiView Universal DAB Detection Kit (Ventana Medical Systems, Tucson, AZ, USA) using the following panel of antibodies: Microtubule Associated Protein 2 (MAP2, clone C, mouse monoclonal, 1:100 dilution, Riederer Lausanne, Waadt, Swiss), Neuronal Nuclei (NeuN, clone A60, mouse monoclonal, 1:1500 dilution, Merck Millipore, Burlington, MA, USA), Kiel 67 protein (Mib1/Ki67, clone SP6/Ki67, rabbit monoclonal, 1:200 dilution, Cell Marque, Rocklin, CA, USA), glial fibrillary acid protein (GFAP, clone 6F2, mouse monoclonal, 1:500 dilution, Dako, Santa Clara, CA, USA), Synaptophysin (clone SP11, 1:100 dilution, Thermo Scientific, Waltham, MA, USA), histone 3 (*H3*) lysine27-to-methionine (*K27M*) mutation (H3K27M, rabbit polyclonal, 1:100 dilution, Merck Sigma-Aldrich^®^, Darmstadt, Germany), isocitrate dehydrogenase-1 (IDH1, clone H09, mouse monoclonal, 1:50 dilution, Dianova, Eching, Bavaria, Germany), tumor protein p53 (p53, clone D0-7, mouse monoclonal, 1:2000 dilution, Dako, Santa Clara, CA, USA), Cyclin-dependent kinase inhibitor 2A (p16, clone G175-405, mouse monoclonal IgG1, BD Bioscience, Franklin Lakes, New Jersey, USA) and cluster of differentiation 34 (CD34, clone QBEnd-10, mouse monoclonal, 1:100 dilution, Dako, Santa Clara, CA, USA). Both reviewers unanimously achieved a histopathology diagnosis using the WHO classification of tumors of the CNS valid at the time of diagnosis.

### Whole-exome sequencing (WES) and copy-number variation (CNV) analysis

Two of the senior authors and experienced neuropathologists, RC and IB, histopathologically confirmed the presence of lesional tumor cells in all frozen tissue samples (*n* = 72, Table 1). Following routine DNA extraction from the frozen tissue samples, whole-exome sequencing (WES) was performed at a coverage of > 350 × using Agilent SureSelect Human All Exon V7 enrichment and paired-end reads (151 bp) Illumina sequencing. Paired-end FASTQ files were pre-processed following GATK best practices [13] and genetic variants were identified as described previously [23]. These samples were also genotyped using SNP analysis (see above). The resulting single nucleotide polymorphism data was used to detect somatic CNV as described previously [23]. Here, genetic variants were defined as either mutation, CNV or SNP. The identified genetic variants were visualized with semi-automatically generated oncoplot graphics using Excel sheets 2016 oncoplot template (GitHub: https://github.com/ptgrogan/excel-oncoplot). The oncoplot was then sorted by genetic variants in the RAS-/MAP-Kinase signaling pathway to compare with histopathology features and clinical outcome data.

### DNA methylation array processing and CNV-calling

DNA was extracted from 84 FFPE tissue blocks histopathologically reviewed by RC and IB to confirm the presence of lesional cells and using the QIAamp DNA Micro Kit (Qiagen, Venlo, Netherlands) following the manufacturer’s routine protocol (Table 1, *n* = 28 and supplemental table, online resource, *n* = 56). Methylation profiling was performed with the Infinium HumanMethylation450K in 19 samples or Infinium MethylationEPIC 850 k BeadChip in 65 samples (Illumina, San Diego, CA, USA). Twenty-eight cases had both, WES/SNP and DNA methylation analyses (Table 1). In this study, we also included previously published methylation array data of 168 cases publically available from Capper et al. 2018 [8], and 26 cases by Wefers et al. 2020 [39].

We performed differential DNA methylation analysis using a self-customized Python wrapped cross R package pipeline as previously described [19] and publicly available at https://github.com/FAU-DLM/Methylr2py. Methylation data from 850K and 450K data were combined into a virtual array with the ‘combineArrays’ function of the minfi package [1]. We stratified quantile normalized data using the ‘minfi’ ‘preprocessQuantile’ function [15]. Probes targeting sex chromosomes, containing single-nucleotide polymorphisms, not uniquely matching, and known cross-reactive probes were removed [11]. As a result, 128,525 probes were included in the combined virtual array and used for further analysis. Uniform Manifold Approximation and Projection (UMAP) for general non-linear dimensionality reduction was used for visualization [26] and to find subgroups compared to previously published LEAT cases [8]. The following non-default parameters were used: init = random, min_dist = 0.0, spread = 3.0. To confirm the identified clusters from the previous step, we applied unsupervised learning using HDBSCAN as a clustering algorithm [25]. The following non-default parameters were used herein: min_samples = 4, min_cluster_size = 4. Subsequent copy number calling was performed with ‘conumee’ Bioconductor package v.1.28.0. [17]. To be able to perform CNV summary plots of identified clusters from the HDBSCAN, we adopted and extended the ‘conumee’ package functionality. The new functions enabling summary plots from the ‘conumee’ package are forked from the original project and publicly available at https://github.com/FAU-DLM/conumee.

## Results

### Whole exome sequencing and CNV detection

Seventy-two tissue samples were submitted to WES. Fourty-four samples carried a genetic variant (61%), i.e., mutations, gains in copy number variants (CNV), or loss of heterozygosity (LOH) and were further studied herein (Fig. 1). Our histopathology review confirmed the diagnosis of ganglioglioma CNS WHO grade 1 regarding histological and immunohistological criteria specified by the WHO in 70 samples. One case was compatible with the diagnosis of a multinodular and vacuolating tumor (MNVT; case #14) and one case was histopathologically compatible with a collision of GG with Pleomorphic Xanthoastrocytoma (PXA; case #20). Quality assessment and significance levels of the genomic data were determined as described previously [23].

We observed three major patterns of genetic abnormalities in this cohort. Group 1 consisted of 15 cases with complex genetic variants (CGV) and in parallel with several aberrations in the RAS/MAP-Kinase and PI3K/AKT/MTOR pathways. Eight samples had alterations in the *PTPN11* gene (53%), seven with CNV gains and one with a missense variant. These eight samples frequently had also mutations in *BRAFV600E* (*n* = 7) and common CNV gains in *KRAS* (*n* = 7), *NF1* (*n* = 7), *FGFR2*, *3 or 4* (*n* = 6). Less frequent CNV gains were observed in *BRAF* (*n* = 5), *RHEB* (*n* = 5), and other RAS-/MAPK-pathway genes (*n* = 4). The remaining seven samples of this group of GG with complex and multiple hits all had a *BRAFV600E* mutation and/or *BRAF* gains plus additional hits in *FGFR2, 3 or 4* (*n* = 6, 86%), *RHEB* (*n* = 5, 71%), *CBL* (*n* = 2, 29%), or *SHOC2*, *MEK2*, *NF1*, (*n* = 1 for each), *CDKN2A* LOH (*n* = 1) and other PI3K/AKT/MTOR pathway genes (*n* = 6, 86%). None of these cases revealed *H3K27M* alterations when using WES/SNP-array analysis nor immunohistochemistry. Predisposing germline alterations were not found in any of these samples.

Group 2 contained 29 tissue samples characterized by a *BRAFV600E* mutation (*n* = 28; 97%) or other non-complex genetic alterations. Three samples had a *BRAFV600E* mutation and a CNV loss in *BRAF*, a *PIK3CA* missense variant, or a gain in *CBL* and *MEK2*. One sample only had LOH of *NPRL2* (Fig. 1). The remaining 28 cases of our cohort of GG submitted to WES revealed no genetic alterations discernable by our techniques, i.e., Group 3. However, we cannot rule out the possibility of incomplete sampling with low amounts of pathological cellular content in the DNA sample.

### DNA methylation analysis

DNA methylation array data were obtained from 84 patients in our patient series (Table 1 and supplemental table, online resource). In addition, we retrieved 194 reference samples from published sources to apply UMAP analysis and unsupervised clustering using the HDBSCAN methods. Additional copy-number profiling was performed utilizing the extended ‘conumee’ package for all samples mentioned above (see Material and Methods; https://github.com/FAU-DLM/conumee). UMAP and HDBSCAN analysis depicted readily expected and established methylation classes for the reference cohort. Twenty-one of our samples were also assigned to these established groups (Fig. 2; Table 1 and supplemental table, online resource). This reference cohort consisted of Ganglioglioma (LGG, GG), Dysembryoplastic Neuroepithelial Tumours (LGG, DNT), Pleomorphic Xantoastrocytoma (LGG, PXA), and diffuse astrocytoma, MYB- or MYB-L1 altered (LGG, MYB). However, we detected a fifth, new methylation class, including all samples of our series with complex genetic variants (Fig. 1), including those with alterations in *PTPN11/KRAS/NF1,* and henceforth labeled as GG, PTPN11. The “GG, PTPN11 methylation class” consisted of a total of 63 of the 84 cases. They were histopathologically diagnosed as ganglioglioma (*n* = 56), ganglioglioma with increased proliferation rate analog WHO II° (*n* = 1), anaplastic ganglioglioma WHO III° (n = 1), or PXA (*n* = 5).

### Copy number profiling from DNA methylation array analysis

**Fig. 3:**
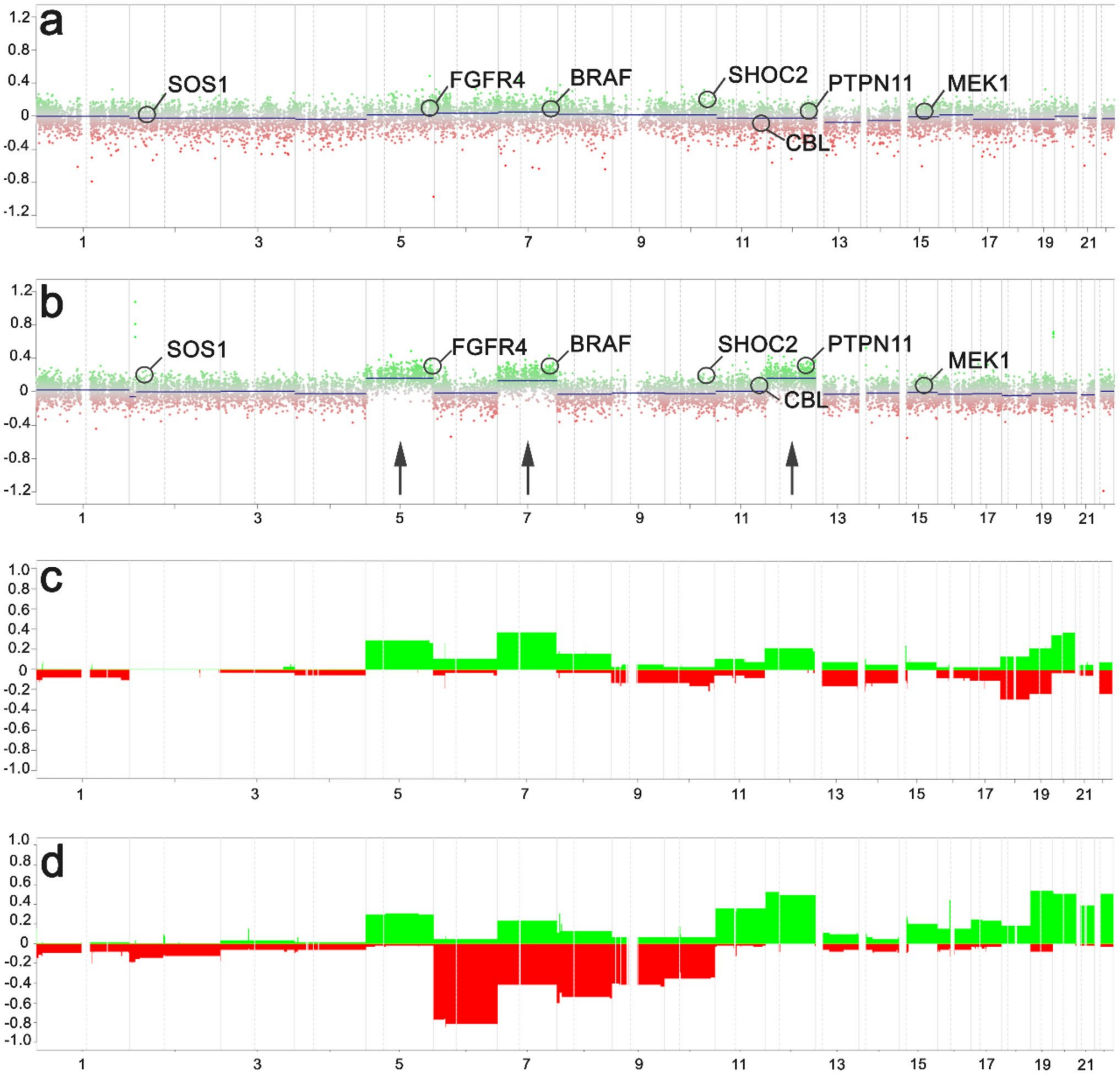
Copy number profile of different methylation groups. While the published reference cohort of ganglioglioma showed a flat profile with marginal gains at chromosomes 5, 7 and 12 in **a** and **c** (c represents a summary plot of the entire LGG, GG cohort, *n* = 38), frequent gains and losses were evident within the new ganglioglioma methylation class with adverse outcome as shown in **b** (patient sample #7, see Table 1) and **d** (summary plot of entire GG, PTPN11 cohort, *n* = 63). Notably, 31 of 63 tumors of this new methylation class showed gains on the long arm of chromosome 12, including *PTPN11*. X-axis list chromosomes; Y-axis indicates log(R) ratio in **a** and **b**, and the percentage of samples showing an alteration in **c** and **d**. Gains are labeled in green, losses in red

We performed an additional analysis of copy number calling from 450 and 850 k array data using the ‘conumee’ package to confirm the SNP-based CNV detection. Typical GG of the DNA methylation class LGG, GG showed a flat CNV profile with marginal gains at chromosomes 5, 7 and 12 (Fig. 3), compatible with RAS-/MAP-kinase pathway alterations described in this diagnostic entity [7]. In contrast, the new methylation class comprising GG with complex genomic alterations and an adverse postsurgical outcome, i.e., GG with adverse clinical outcome, showed frequent gains at chromosomes 5, 7, and 12, as well as losses at chromosomes 6, 7, 8, and partially of chromosome 9 (Fig. 3b, d).

### Genotype–phenotype correlation

**Fig. 4:**
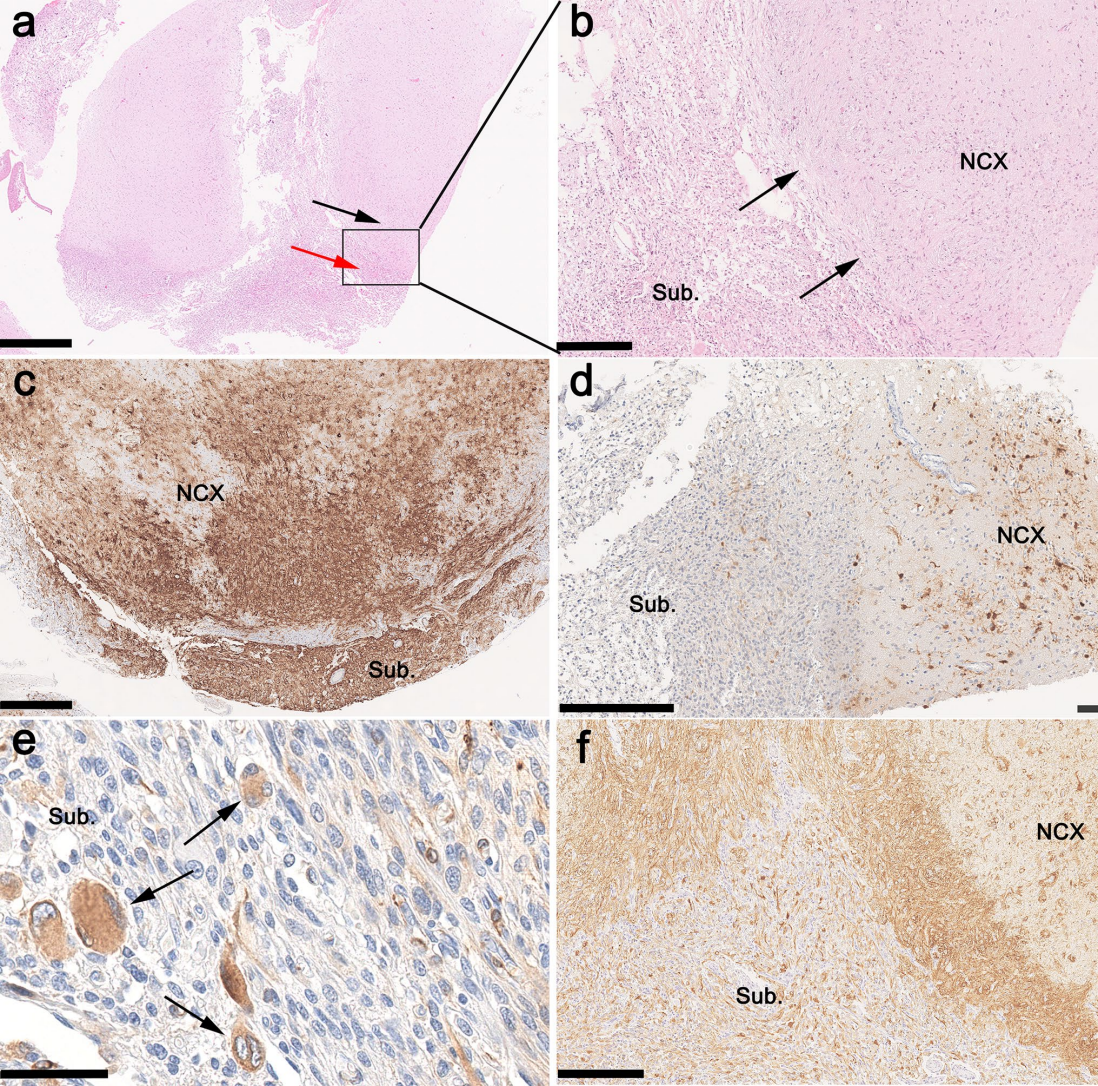
Histopathology findings in *PTPN11/KRAS/NF1* altered Ganglioglioma**.** A representative case of *PTPN11/KRAS/NF1* altered ganglioglioma (case #8; Table 1). Upper row, **a** and **b** Intra- and extracortical tumor growth (black and red arrow in **a**) was readily visible on HE staining. Arrows in **b** indicate the border of the cortical surface and subarachnoidal space. CD34-immunoreactivity (**c**) was a prominent feature of cortical and subarachnoidal tumor growth. In contrast, p16-immunoreactivity (**d**) was accentuated in the cortical tumor component (subarachnoidal component on the left). Multinucleated cells (**e**) in the subarachnoidal space, immunoreactive for MAP2, were classified as dysplastic neurons (arrows). The astroglial component was predominant (**f**, GFAP immunohistochemistry). NCX—neocortex, Sub—subarachnoidal space. Scale bars: **a** 1 mm; **b, d, f** 250 µm; **c** 500 µm; **e** 100 µm

We further assessed the histopathology phenotype of eight GG with *PTPN11/KRAS/NF1* alterations in our cohort as identified and confirmed by the above mentioned molecular-genetic studies. These tumors were characterized by a glio-neuronal phenotype (Fig. 4). The neoplastically transformed glial cell population shared features of astroglia including prominent immunoreactivity with antibodies against GFAP (Fig. 4f). Oligodendroglia-like cell features were not dominant in these tumors. A dysplastic neuronal cell component was another hallmark, some of which were located in the subarachnoidal space (Fig. 4e), containing multiple nuclei (Fig. 4e) and being immunoreactive for MAP2 (Fig. 4e) and synaptophysin (not shown). Tumor growth was mostly diffuse into the grey and white matter (Fig. 4b), but partially also nodular. Interestingly, seven tumors with *PTPN11/KRAS/NF1* alterations (Table 1) revealed growth into the subarachnoidal space (supplemental Fig. 1, online resource). Within the new methylation class of 63 GG with adverse clinical outcome (GG, PTPN11), histopathology analysis revealed (i) subarachnoidal growth in 42 cases (67%, Fig. 4b and supplemental Fig. 1, online resource), (ii) multinucleated giant cells in 16 (25%; Fig. 4e), (iii) white matter rarefaction and diffuse growth in 12 (19%), and (iv) multinodular growth within the neocortex in 6 (9.5%, supplemental Fig. 1, online resource). However, 18 cases shared more than one of these atypical histopathology features. A cohort of 8 cases did not have sufficient tissue materials and/or anatomical landmarks to firmly support a microscopic conclusion. Immunohistochemistry was most helpful in recognizing the complex growth pattern of these tumors, which were immunoreactive for CD34 and p16 in all cases (Fig. 4c and d). In contrast to p16, CD34 was consistently seen also in the subarachnoidal space. There was no evidence for mutant *IDH1 R132H* or *H3K27M* labeling nor an increased proliferation activity above 5%. Some specimens also showed small calcifications.

*PTPN11/KRAS/NF1*-altered tumors accounted for 53% of tumors with complex genetic variants in our WES cohort and 49% of tumors in the methylation class GG, PTPN11 with adverse clinical outcome. Interestingly, five out of eight patients with *PTPN11/KRAS/NF1* altered GG were not seizure-free after surgery. Targeted therapy with small molecule inhibitors of the MAPK-pathway was not applied in any case, however. Furthermore, one patient died of sudden unexpected death in epilepsy (SUDEP; Table 1). The low seizure freedom rate of 38% (Engel class I) in this cohort contrasts with the favorable outcome in 76% of patients in group 2 with GG characterized just by *BRAFV600E* mutations or in 72% of patients of group 3. This holds true also when statistically comparing group 1 with group 2 (Chi-square *p* = 0.007; Yates correction *p* = 0.018). Sufficient clinical data within group 3 of GG without any genetic variant detectable by our described methodology was available in 22 out of 27 cases. One patient of group 3 had a provoked seizure (alcohol + sleep deprivation; case 47) and another had a partial resection (case 46).

## Discussion

Our study identified a new DNA methylation class (MC) consisting of 63 cases of patients with LEAT submitted to epilepsy surgery due to intractable focal seizures. All eight *PTPN11/KRAS/NF1-*altered GG recognized in our previous study [23] were assigned to the novel MC by the algorithm and can be characterized by (1) subarachnoidal growth of biphasic, glio-neuronal cells, (2) complex brain somatic gene variants in the RAS-/MAP-Kinase and PIK3–AKT/mTOR pathways, (3) predominant localization in the temporal lobe, and (4) adverse postsurgical outcome, i.e., not being seizure-free. Almost half of the 63 cases assigned to the new MC had copy number gains on the long arm of chromosome 12, including the *PTPN11* locus as recognized by our newly extended ´conumee´ CNV profiling now publically available on GitHub. However, the adverse post-surgical seizure outcome warrants further analysis in larger and prospectively collected tumor cohorts as patients will require adjunct medical therapies to prevent the harmful consequences of an active, long-term epilepsy. Yet, the histopathology surrogate markers described herein will not allow us to reliable recognize all tumors in this LEAT subgroup at the microscopy level. This supports the implementation of an integrated genotype–phenotype diagnosis also for LEAT.

The histopathological classification of LEAT remains an ever-challenging issue [3, 5, 33]. This applies in particular to the variable phenotypes of GG due to more or less dominant (i) neoplastically transformed astroglia, ii) neoplastically transformed oligodendroglial-like cells and (iii) a dysplastic neuronal cell population [5]. Subarachnoidal growth and adverse outcome in our tumor cohort suggested the differential diagnosis of PXA. However, neither the histopathology phenotype, e.g., the absence of reticulin fibers, xanthomatous cells, and immunoreactivity for p16, nor the absence of homozygous deletions of CDKN2A/B supported this diagnosis in our cohort (Figs. 3 and 4). In addition, DNA methylation profiling readily separated the class of GG with adverse clinical outcome from PXA (Fig. 2). Several publications also describe composite tumors of variable entities, most often including GG and PXA, a feature in need of further and systematic molecular–genetic exploration in the LEAT cohort [24, 38].

As proven for many high-grade gliomas and embryonal brain tumours described in the 5th edition of the WHO classification scheme, comprehensive genotype–phenotype studies are likely to resolve the issue of interrater reliability and agreement, when based on robust clinical data. We applied genome-wide deep sequencing combined with CNV and DNA methylation array analysis and microscopy studies of 72 snap-frozen GG obtained from patients with careful clinical characterization and postsurgical follow-up to address this issue. Interestingly, our study design identified a cohort of 63 GG as a novel DNA methylation class containing samples not recognizable with data used to build the Heidelberg classifier (Fig. 3). *PTPN11/KRAS/NF1-*altered GG with adverse clinical outcome accumulated in this class and which was provisionally termed herein as *GG, PTPN11*.

**Fig. 5:**
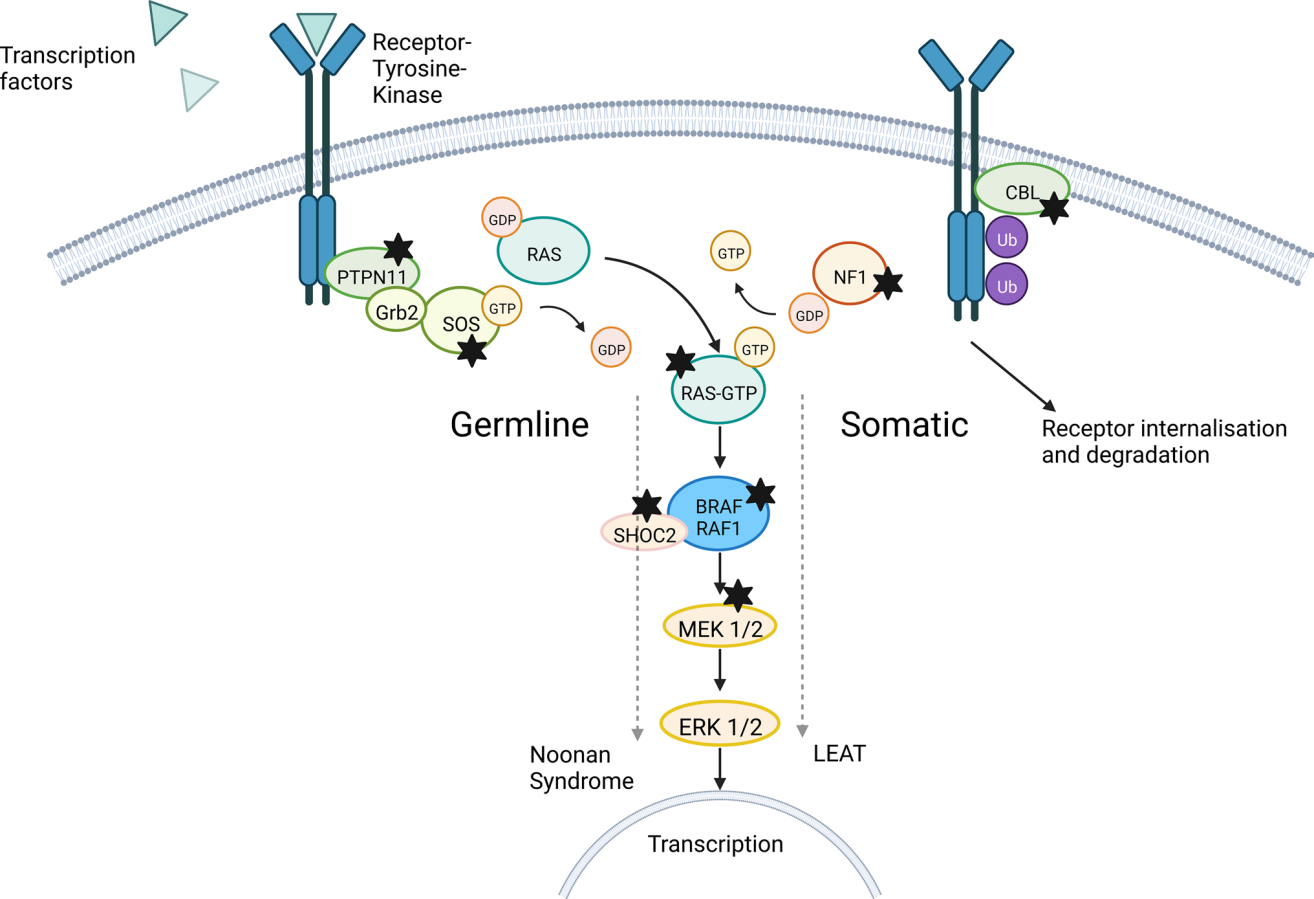
RAS-/MAP-Kinase pathway alterations in GG with adverse clinical outcome. Germline alterations of *PTPN11* and other *RAS-/MAP*-kinase-pathway genes were known to cause Noonan-Syndrome [27, 30, 32]. Genes marked with a black star showed brain somatic alterations in our cohort of GG

*PTPN11* was previously identified as a novel epilepsy gene [2, 18, 23]. However, it was never described before in brain somatic epilepsy-associated disorders. Germline alterations of *PTPN11* are associated with Noonan syndrome (NS, Fig. 5), the most frequent dysfunctional growth syndrome. Neuroepithelial tumors also occur in patients with NS as does focal epilepsy [22, 30, 32, 35]. Other members of the RAS-/MAP-kinase pathway affected within our series of GG with adverse clinical outcome were also known to contribute to Noonan-associated syndromes, e.g., *KRAS, SHOC2*, *CBL* or *MEK1* [27, 30, 32]. In our cohort they either revealed a gain of function mutation or a gain of copy number at the level of brain mosaicism [23]. Experimental evidence demonstrated that qualitatively and quantitatively increased activity of SHP2 causally leads to increased neurogenesis and defective migration behavior of neurons. In addition, the maturation of glial cells via the JAK/STAT pathway was negatively influenced [16]. However, a causative context for *PTPN11* in GG is experimentally not yet confirmed. On the other hand, there is ample evidence for the role of *BRAFV600E* mutations in GG. *In-utero* electroporation of *BRAFV600E* into the developing mouse brain cause CD34-immunoreactive GG and focal epilepsy [20]. This finding aligns with our second cohort of 26 patients with GG and *BRAFV600E* (Fig. 1). However, our cohort of GG with adverse clinical outcome also showed alterations in the PI3K–AKT/MTOR pathway frequently involved in epileptogenic Focal Cortical Dysplasia ILAE Type 2 [4]. Another newly described experimental GG mouse model using a combination of *BRAFV600E* with mTOR pathway alterations would thus be an intriguing option to further explore the nature of the new GG category [9]. In depth microscopic inspection of all our tumor samples did not show evidence for any associated FCD according to the ILAE classification scheme of 2022, i.e. FCD ILAE Type 3b. Nevertheless, we cannot exclude that malformations of the neocortex may occur in non-resected parts in addition to tumor satellites and thus lead to a worse postoperative outcome.

It is difficult to reconcile the failure of seizure freedom following epilepsy surgery in our retrospective and small patient cohort. Subarachnoidal tumor growth might contribute to the incompleteness of surgical resection, particularly when not readily anticipated from pre-surgical neuroimaging studies. Another option is a larger tumor area not visible on MRI and not included in the surgical field as indicated by the diffuse infiltration pattern of CD34- and p16-immunoreactive tumor satellites. The impact of transcriptional signatures in LEAT reflecting the clinical outcome has also been recently postulated [12]. This work distinguished three subgroups of GG, i.e., *BRAF*-altered GG, juvenile GG, and not otherwise specified. Interestingly, the *BRAF*-altered group of GG was characterized by transcriptional changes in RAS-/MAPK-pathway including the *FGFR* genes. These cases also had a “progressive disease” after surgery including tumor relapse/recurrence and were postulated to aim for gross-total resection to prevent patients from tumor recurrence. However, the epileptogenic tumor network can also be compromised by other cell biology features, e.g., the microtubular glioma network, which is not yet studied nor anticipated well in LEAT [29, 36, 37].

Lack of seizure-freedom significantly impacts the quality of life and patient survival, i.e., sudden unexpected death in epilepsy (SUDEP) [14]. Many studies indicate that the overall mortality rate for people with epilepsy is elevated two- or threefold compared to the general population [28]. Indeed, our patient #8 (Table 1, Fig. 1) died from SUDEP 2 years after a seizure relapse from an otherwise successful surgical approach. Such adverse clinical features are not recognized in the WHO grading scale, which only addresses the risk of tumor recurrence and/or malignant progression. This contrasts routine clinical practice in the realm of epileptology and epilepsy surgery, in which seizure freedom is of utmost importance for patient survival and quality of life, in particular for the group of LEAT with their favorable outcome prediction measures in general [6, 21]. The advancement of comprehensive genotype–phenotype association studies may reinforce the discussion and communication across medical disciplines to help developing the best available disease classification schemes and reflecting and taking into account all relevant disease parameters.

As a matter of fact, integrated genotype–phenotype classification schemes increasingly govern targeted medical therapies. Patients’ access to tailored epilepsy surgery will remain, however, of utmost importance. Tumour tissue samples will then help to reliably identify a tumour subtype and possible molecular targets when surgery had failed. Indeed, a variety of targeted therapies were already developed for the gene product of the *PTPN11*, such as orthosteric inhibition of the *SHP2* protein [34].

## Supplementary Information

Below is the link to the electronic supplementary material.

Supplementary file1 (DOCX 12753 KB)

## Data Availability

The complete methylation data of the 86 samples included in this study, will be deposited in NCBI’s Gene Expression Omnibus (GEO, http://www.ncbi.nlm.nih.gov/geo). The accession number is GSE218542.
