# Correction to: Ganglioglioma with adverse clinical outcome and atypical histopathological features were defined by alterations in *PTPN11/KRAS/NF1* and other RAS-/MAP-Kinase pathway genes

**DOI:** 10.1007/s00401-023-02577-x

**Published:** 2023-04-28

**Authors:** Lucas Hoffmann, Roland Coras, Katja Kobow, Javier A. López-Rivera, Dennis Lal, Costin Leu, Imad Najm, Peter Nürnberg, Jochen Herms, Patrick N. Harter, Christian G. Bien, Thilo Kalbhenn, Markus Müller, Tom Pieper, Till Hartlieb, Manfred Kudernatsch, Hajo Hamer, Sebastian Brandner, Karl Rössler, Ingmar Blümcke, Samir Jabari

**Affiliations:** 1 Department of Neuropathology, Partner of the European Reference Network (ERN) EpiCARE, Universitätsklinikum Erlangen, FAU Erlangen-Nürnberg, Erlangen, 91054 Germany; 2 Department of Molecular Medicine, Cleveland Clinic Lerner College of Medicine, Case Western Reserve University, Cleveland, USA; 3 Genomic Medicine Institute, Lerner Research Institute, Cleveland Clinic, Cleveland, OH 44195 USA; 4 Charles Shor Epilepsy Center, Neurological Institute, Cleveland Clinic, Cleveland, USA; 5 Stanley Center for Psychiatric Research, Broad Institute of Harvard and M.I.T, Cambridge, MA 02142 USA; 6 Cologne Center for Genomics (CCG), Medical Faculty of the University of Cologne, University Hospital of Cologne, 50931 Cologne, Germany; 7 Center for Neuropathology and Prion Research, LMU Munich, Munich, Germany; 8 Department of Epileptology (Krankenhaus Mara), Medical School, Bielefeld University, Bielefeld, 33617 Germany; 9 Center for Pediatric Neurology, Neurorehabilitation, and Epileptology, Schoen-Clinic, Vogtareuth, 83569 Rosenheim, Germany; 10 Epilepsy Center, EpiCARE Partner, Universitätsklinikum Erlangen, FAU Erlangen-Nürnberg, Erlangen, 91054 Germany; 11 Department of Neurosurgery, EpiCARE Partner, Universitätsklinikum Erlangen, FAU Erlangen-Nürnberg, Erlangen, Germany; 12 Department of Neurosurgery, EpiCARE Partner, Medical University of Vienna, Vienna General Hospital, Vienna, Austria; 13 Department of Neurosurgery (Evangelisches Klinikum Bethel), Medical School, Bielefeld University, Bielefeld, 33617 Germany

**Correction to: Acta Neuropathologica** 10.1007/s00401-023-02561-5

In the original publication, incorrect version of Fig. 3 and wrong format of Table 1 were published. Specifically, the labels in Fig. 3 were missing and columns of Table 1 were not in the right format.

The correct versions of Fig. 3 and Table 1 are shown below.

**Fig. 3:**
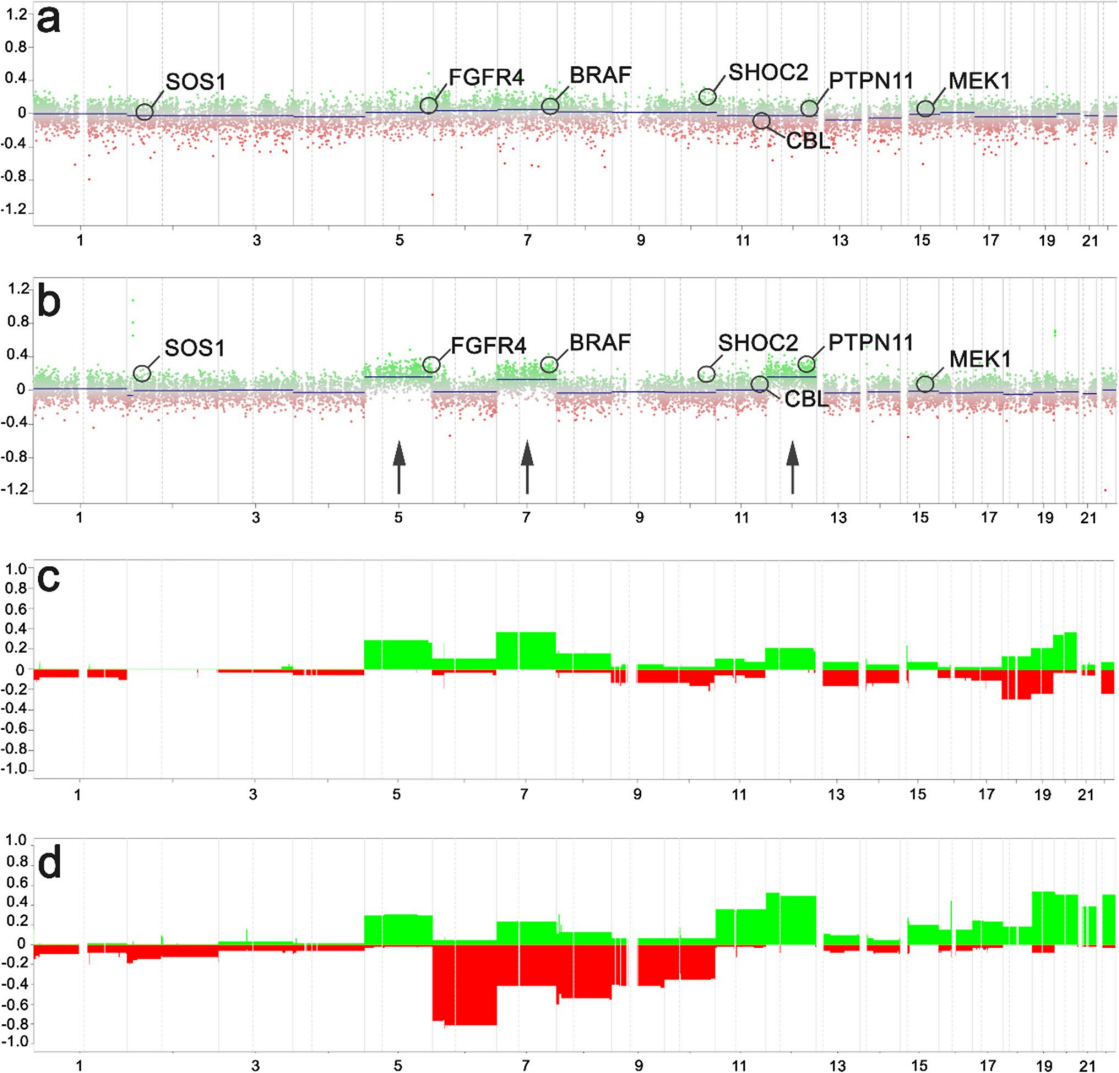
Copy number profile of different methylation groups. While the published reference cohort of ganglioglioma showed a flat profile with marginal gains at chromosomes 5, 7 and 12 in 3 (**a** and** c**) (**c** represents a summary plot of the entire LGG, GG cohort, *n* = 38), frequent gains and losses were evident within the new ganglioglioma methylation class with adverse outcome as shown in **b** (patient sample #7, see Table 1) and **d** (summary plot of entire GG, PTPN11 cohort, *n* = 63). Notably, 31 of 63 tumors of this new methylation class showed gains on the long arm of chromosome 12, including *PTPN11*. *X*-axis list chromosmes; *Y*-axis indicates log(*R*) ratio in **a** and **b**, and the percentage of samples showing an alteration in **c** and **d**. Gains are labeled in green, losses in red

**Table 1:** Summary of 72 patients with WES and SNP, 28 of which also had DNA methylation analysis

ID	GTT	Outcome	FU	DoE	Onset	Sex	DX	MC	OG
1	SNP, WES, 850k	Engel 4	4	2	N/A	M	GG 1	GG, PTPN11	Complex Genetic Variant
2	SNP, WES, 850k	Engel 1a	2	2	N/A	M	GG 1	GG, PTPN11	
3**	SNP, WES, 450k	Engel 1b	2	4	11–15	M	GG 1	GG, PTPN11	
4	SNP, WES, 850k	Engel 2b	2	40	11–15	F	GG 1	GG, PTPN11	
5	SNP, WES, 850k	Engel 1a	2	4	6–10	M	GG 1	GG, PTPN11	
6	SNP, WES, 850k	Engel 1a	2	1	11–15	F	GG 1	GG, PTPN11	
7	SNP, WES, 850k	Engel 1c	2	2	16–20	F	GG 1	GG, PTPN11	
8	SNP, WES, 850k	Engel 2d SUDEP	2	13	16–20	F	GG 1	GG, PTPN11	
9	SNP, WES	Engel 1b*	6	4	6–10	F	GG 1		
10	SNP, WES, 850k	Engel 1b	3	4	16–20	F	GG 1	GG, PTPN11	
11	SNP, WES, 850k	Engel 1a	2	3	11–15	M	GG 1	GG, PTPN11	
12**	SNP, WES	Engel 1c	2	5	16–20	F	GG 1		
13	SNP, WES, 850k	Engel 1a	2	0.3	6–10	M	GG 1	GG, PTPN11	
14	SNP, WES	Engel 2b	2	45	0–5	M	MNVT		
15	SNP, WES	N/A	N/A	N/A	N/A	M	GG 1		
16	SNP, WES	Engel 1a	2	1.75	0–5	M	GG 1		Non-complex Variant
17	SNP, WES	Engel 1a	2	8.75	0–5	M	GG 1		
18	SNP, WES	N/A	N/A	N/A	N/A	F	GG 1		
19	SNP, WES, 850k	Engel 1a	2	13	6–10	M	GG 1	GG, PTPN11	
20	SNP, WES	Engel 1a	2	5	11–15	M	PXA/ GG		
21	SNP, WES	Engel 1a	2	No seizures		F	GG 1		
22	SNP, WES	Engel 1a	2	5	0–5	M	GG 1		
23	SNP, WES	Engel 1a	2	7	11–15	M	GG 1		
24	SNP, WES	Engel 1a	2	3	0–5	M	GG 1		
25	SNP, WES	Engel 1a	2	9	6–10	F	GG 1		
26	SNP, WES	Engel 1a	2	1	6–10	M	GG 1		
27	SNP, WES	Engel 1a	2	3	16–20	M	GG 1		
28	SNP, WES	Engel 1a	2	7	6–10	F	GG 1		
29	SNP, WES	Engel 1a	2	9	6–10	F	GG 1		
30	SNP, WES	Engel 1a	2	1	0–5	M	GG 1		
31	SNP, WES, 850k	Engel 1a	2	N/A	N/A	M	GG 1	GG, PTPN11	
32	SNP, WES	Engel 1a	2	3	0–5	F	GG 1		
33	SNP, WES	Engel 1a	2	2.25	0–5	F	GG 1		
34	SNP, WES	Engel 1a	2	6	11–15	M	GG 1		
35	SNP, WES	Engel 1a	2	3	0–5	M	GG 1		
36	SNP, WES, 850k	Engel 1a	2	N/A	N/A	M	GG 1	GG, PTPN11	
37	SNP, WES, 850k	Engel 1a	2	11	11–15	F	GG 1	GG, PTPN11	
38	SNP, WES, 850k	Engel 1a	2	N/A	N/A	M	GG 1	GG, PTPN11	
39	SNP, WES, 850k	Engel 2a	2	6	0–5	M	GG 1	GG, PTPN11	
40	SNP, WES	Engel 1a	2	N/A	N/A	M	GG 1		
41	SNP, WES, 850k	Engel 1a	2	6	0–5	M	GG 1	GG, PTPN11	
42	SNP, WES	Engel 3a	2	2	0–5	M	GG 1		
43*	SNP, WES, 850k	Engel 3a	2	24	0–5	F	GG 1	GG, PTPN11	
44	SNP, WES	Engel 1c	2	6	0–5	F	GG 1		
45*	SNP, WES	Engel 3a	2	8	46–50	F	GG 1		No detected Variant
46*	SNP, WES, 850k	Engel 2b	2	16	16–20	M	GG 1	GG, PTPN11	
47**	SNP, WES, 850k	Engel 3a	2	4	21–25	F	GG 1	GG, PTPN11	
48	SNP, WES, 850k	Engel 1b	2	11	11–15	M	GG 1	GG, PTPN11	
49	SNP, WES, 850k	Engel 1b	2	30	0–5	F	GG 1	GG, PTPN11	
50	SNP, WES, 850k	Engel 1b	2	6	0–5	M	GG 1	GG, PTPN11	
51	SNP, WES	Engel 1a	2	4	6–10	M	GG 1		
52	SNP, WES	Engel 1a	2	5	6–10	F	GG 1		
53	SNP, WES	Engel 1a	2	14	11–15	M	GG 1		
54	SNP, WES	Engel 1a	2	0	0–5	M	GG 1		
55	SNP, WES	Engel 1a	2	13	26–30	M	GG 1		
56	SNP, WES, 850k	Engel 1a	2	12	26–30	M	GG 1	GG, PTPN11	
57	SNP, WES	N/A	N/A	N/A	N/A	F	GG 1		
58	SNP, WES, 450k	N/A	N/A	N/A	N/A	M	GG 1	LGG, GG	
59	SNP, WES	N/A	N/A	N/A	N/A	M	GG 1		
60	SNP, WES	Engel 1a	2	24	0–5	F	GG 1		
61	SNP, WES	Engel 1a	2	2	6–10	M	GG 1		
62	SNP, WES	Engel 1a	2	0.25	0–5	F	GG 1		
63	SNP, WES	Engel 1a	2	2	0–5	F	GG 1		
64	SNP, WES	Engel 1a	2	4	0–5	M	GG 1		
65	SNP, WES	N/A	N/A	N/A	N/A	F	GG 1		
66	SNP, WES	Engel 1a	2	2	11–15	F	GG 1		
67	SNP, WES, 850k	Engel 1a	2	0.5	6–10	F	GG 1	GG, PTPN11	
68	SNP, WES	N/A	N/A	N/A	N/A	M	GG 1		
69	SNP, WES	Engel 1a	2	4	0–5	F	GG 1		
70	SNP, WES	Engel 1a	2	N/A	N/A	M	GG 1		
71	SNP, WES	Engel 1a	2	6	6–10	F	GG 1		
72	SNP, WES, 850k	Engel 1a	2	0.25	6–10	M	GG 1	GG, PTPN11	

ID = case numbers, GTT = Genetic testing tool using either Single Nucleotide Polymorphism (SNP), Whole-Exome Sequencing (WES), 850 k or 450 k DNA methylation arrays; Outcome = most recent postsurgical outcome, i.e. seizure freedom, according to Engel [21]; N/A = not available; Follow-up (FU) in years; DoE. = Duration of Epilepsy; Onset = disease onset (age in years); Sex: F—female, M = male; DX = histopathology diagnosis: Ganglioglioma CNS WHO grade 1 (GG 1), Ganglioglioma analogue CNS WHO grade 2 (GG 2), Ganglioglioma analogue CNS WHO grade 3 (GG 3), composite pleomorphic xanthoastrocytoma with ganglioglioma (PXA/ GG), multinucleated vacuolated tumor (MNVT). MC—methylation classes: Ganglioglioma with adverse clinical outcome (GG, PTPN11), Ganglioglioma (LGG, GG), Pleomorphic Xantoastrocytoma (LGG, PXA), (also see Fig. 2); Oncoplot Group (OG) = genetic subgroups from WES/SNP-array (see also Fig. 1). Sixty-two cases were located in the temporal lobe, cases #10, #17, #23, #43 and #50 in the parietal lobe, cases #41, #46 and #67 in the frontal lobe, case #24 in the occipital lobe and case #15 had no localizing data

*Adverse outcome due to incomplete resection

**Patient received a second surgery due to lack of seizure freedom

The original article has been corrected.

