# Supplementary material for: Ganglioglioma with adverse clinical outcome and atypical histopathological features were defined by alterations in PTPN11/KRAS/NF1 and other RAS-/MAP-Kinase pathway genes
Source: Acta Neuropathol. 2023 Mar 27;145(6):815–27. doi: 10.1007/s00401-023-02561-5 (PMC10175344; doi:10.1007/s00401-023-02561-5)
Supplement: Supplementary file 1 — Supplementary file1 (DOCX 12753 KB) [file 401_2023_2561_MOESM1_ESM.docx]

**Supplements**

**Supplemental Table: DNA methylation cohort without available WES/SNP data (n=56)**

**Supplemental Table: continued**

**Legend Supplemental Table:** ID = case numbers, GTT = Genetic testing tool including Single Nucleotide Polymorphism (SNP) or Whole-Exome Sequencing (WES), 850k or 450k DNA methylation arrays; Sex: F – female, M = male; DX = histopathology diagnosis prior to genetic testing: Ganglioglioma CNS WHO grade 1 (GG 1), ganglioglioma analogue CNS WHO grade 2 (GG 2), ganglioglioma analog CNS WHO grade 3 (GG 3), composite pleomorphic xanthoastrocytoma with ganglioglioma (PXA/ GG), dysembryoplastic neuroepithelial tumor CNS WHO grade 1 (DNT 1), isomorphic astrocytoma (isomorphic astro.). MC – methylation classes: Ganglioglioma with adverse clinical outcome (GG, PTPN11), Ganglioglioma (LGG, GG), Dysembryoplastic Neuroepithelial Tumours (LGG, DNT), Pleomorphic Xantoastrocytoma (LGG, PXA), and diffuse astrocytoma, MYB- or MYB-L1 altered (LGG, MYB), Glioblastoma (GBM).

**Supplemental Figure 1: Histomorphology features of GG with adverse postsurgical outcome**

**
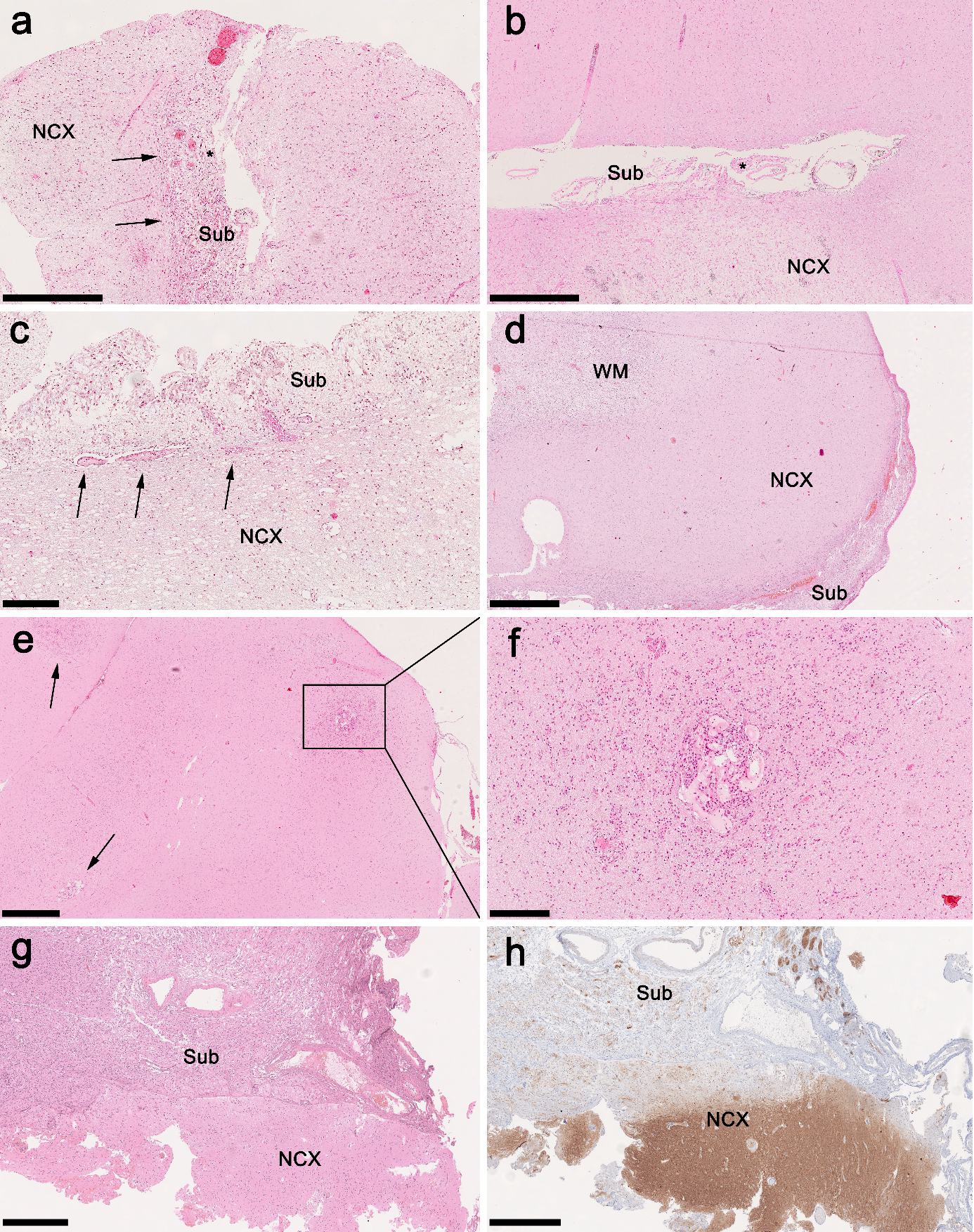
**

**Legend to Supplemental Figure 1:** Six representative cases (H&E: **a:** case #45, **b:** case #11, **c:** case #9, **d:** case #37,**e:** case #5, **g:** case #10, Table 1) of our cohort with lack of postsurgical seizure freedom. Ganglioglioma with subarachnoidal growth pattern (Subarachnoidal – Sub; **a-d**, **g**, **h:** MAP2) and multinodular cortical, partly mucoid (**f**) growth pattern (arrows in **e**, magnification in **f**). Neocortex- NCX; White Matter – WM; arrows in **a** and **c** indicating cortical border with adjacent meningeal vessels. Asterisk (**a**, **b**) indicating subarachnoidal growth into sulci. The subarachnoidal growth pattern was seen in 42 of 63 cases (67%) in GG with adverse clinical outcome. Growth pattern resembling cystic white matter and diffuse cortical growth (see also supplemental Fig. 1) were seen in 12 of 65 cases (18.5%). Scale bars: a, d, g, f: 500µm, b: 1mm, c: 250µm

**Supplemental Figure 2: Histomorphology features of GG with postsurgical seizure freedom**

**
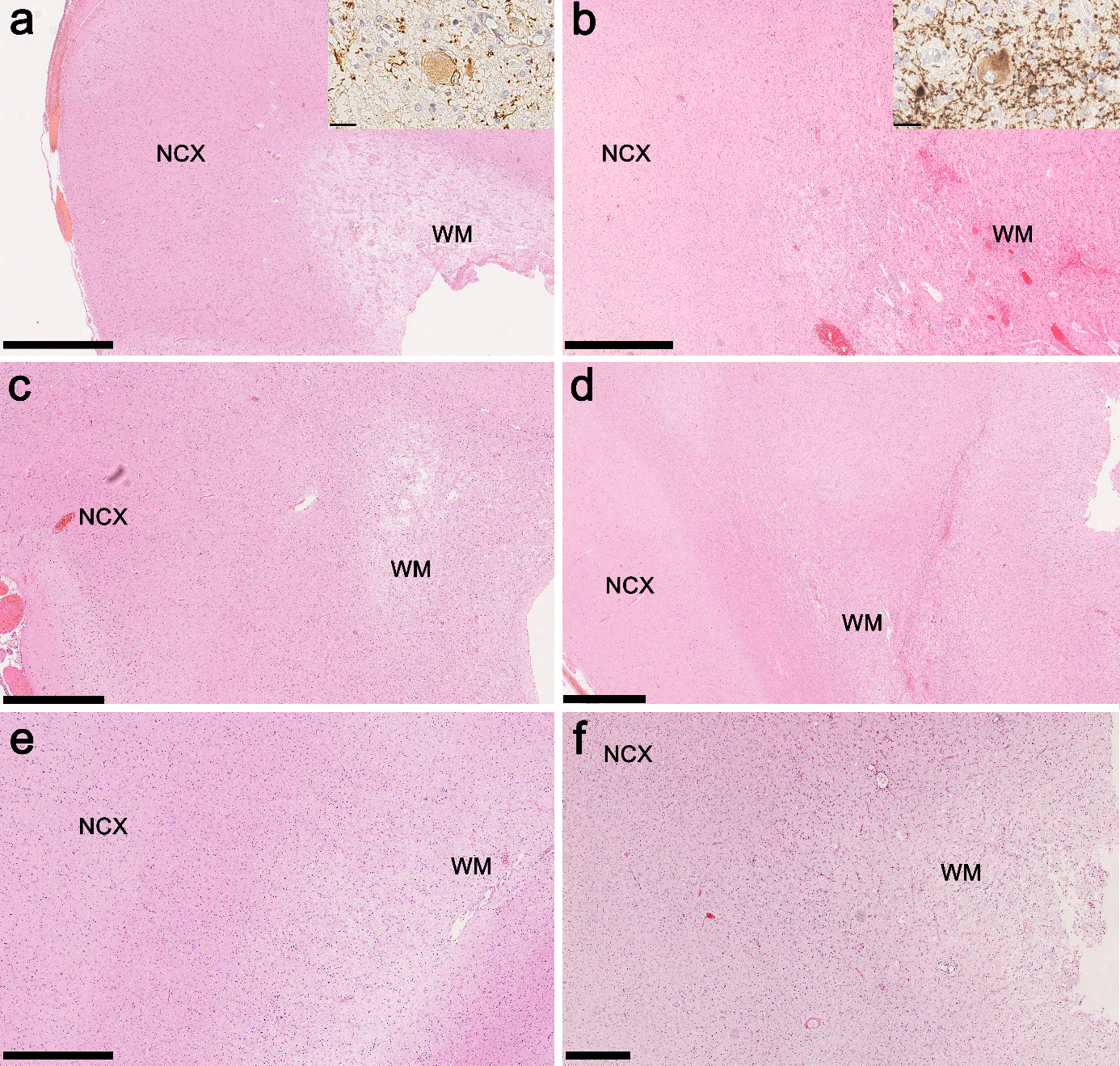
**

**Legend to Supplemental Figure 2:** Five representative GG cases (H&E: **a:** case **#**25, **b:** case #26, **c:** case #112, **d:** case #23,**e:** case #29, **f:** case #22; Supplemental Table 1 & 2) out of our cohort with post surgical seizure freedom. Ganglioglioma (a-f) with cystic white matter rarefaction (WM) and diffuse outgrowth in neocortical (NCX) areas. Neoplastic neurons approved in WM (inlets **a** and **b**; MAP2). Combined cystic white matter and diffuse cortical growth pattern were seen in 34 out of 42 cases (81%) with postsurgical seizure freedom. Subarachnoidal growth (see also Fig. 4 and supplemental Fig. 2 was seen in 2 of 42 cases (≈5%). Scale bars: 1mm, insets: 25µm
